# Evaluation of *in vivo* antibacterial drug efficacy using *Caenorhabditis* *elegans* infected with carbapenem-resistant *Klebsiella pneumoniae* as a model host

**DOI:** 10.3389/fphar.2022.973551

**Published:** 2022-08-19

**Authors:** Huijuan Yao, Ajing Xu, Jingxian Liu, Fang Wang, Huimin Yao, Jihui Chen

**Affiliations:** ^1^ Department of Pharmacy, Xinhua Hospital Affiliated to Shanghai Jiaotong University School of Medicine, Shanghai, China; ^2^ Clinical Laboratory, Xinhua Hospital Affiliated to Shanghai Jiaotong University School of Medicine, Shanghai, China; ^3^ School of Traditional Chinese Medicine, Jilin Agriculture Science and Technology University, Jilin, China

**Keywords:** *Caenorhabditis elegans*, carbapenem-resistant *Klebsiella pneumoniae*, model host, antimicrobial, drug sensitivity testing

## Abstract

**Objective:** This study was developed to assess the *in vivo* antimicrobial activity of specific drugs using a model system consisting of *Caenorhabditis elegans* (*C. elegans*) infected with Carbapenem-resistant *Klebsiella pneumoniae* (CRKP) in an effort to identify promising drugs for CRKP-infected patient treatment.

**Methods:** A *C. elegans*-CRKP liquid assay platform was developed and used to conduct limited *in vivo* screening for antimicrobial agents with potential activity against CRKP. Time curves for 10 different concentrations of tested antimicrobial agents were tested in this model system at 0, 2, 6, 8, and 12 h after treatment. The protective effects of these different antimicrobial agents were compared at different time points. Furthermore, ten CRKP strains samples were isolated from clinical specimens to demonstrate the applicability of the nematode model method, and two typical clinical cases are presented.

**Results:** CRKP bacteria were sufficient to induce *C. elegans* death in a dose- and time-dependent fashion, while effective antimicrobial agents improved the survival of these nematodes in a dose-dependent manner. Notably, PB and TGC exhibited robust antibacterial protection within 12 h even at low tested concentrations, and clear efficacy remained evident for high doses of CAZ at this same time point as mediators of improved nematode survival. The results of *C. elegans* model method were well consistent with that using the Kirby-Bauer method in 10 CRKP strains samples, and two typical clinical cases showed applicability, reliability and efficacy of *C. elegans* model method.

**Conclusion:** Overall, nematode models in drug sensitivity testing have shown advantages in clinical settings. Our results highlight the value of *C. elegans* model systems as tools for the simultaneous screening of different agents for *in vivo* antibacterial efficacy and are deserved further study.

## Introduction

Carbapenem-resistant *Klebsiella pneumoniae* (CRKP) represents a growing threat to global public health as there are few effective treatments for it and it incurs high rates of morbidity and mortality (Agyeman et al., 2020). CRKP bacteria are generally resistant to multiple classes of antibiotics, with only a few drugs including polymyxin B (PB), tigecycline (TGC), aminoglycosides, fosfomycin (FOT), ceftazidime-avibactam, imipenem-cilastatin-relebactam, and meropenem-vaborbactam exhibit favorable *in vitro* activity against CPKP (van Duin et al., 2013; Zhanel et al., 2018; Zhang et al., 2019). These limited therapeutic options make the treatment of infected patients very challenging. The World Health Organization (WHO) has classified CRKP as a critical pathogen that necessitates urgent study and the development of novel efficacious antibiotic therapies (Tacconelli et al., 2017). As such, efforts to treat CRKP represent an active area of clinical research. In clinical settings, the Kirby-Bauer (K-B) disk diffusion method is frequently utilized to detect CRKP drug sensitivity, but this approach is subject to certain technical limitations and is of limited value in the context of high-throughput screening. Moreover, it is not possible to determine whether drugs that exhibit *in vitro* resistance will present with *in vivo* activity. The *Caenorhabditis elegans* (*C. elegans*) model system is a small (∼1 mm long) genetically tractable multicellular organism that has been used in a wide range of research fields for over five decades (Elkabti et al., 2018), offering value in studies of development, neurobiology, and small RNAs (Balla and Troemel, 2013; Pir et al., 2017). These nematodes exhibit a short reproductive cycle, have a fully sequenced genome, and can be readily infected with pathogenic bacteria such as *Pseudomonas aeruginosa*, *Staphylococcus aureus*, *Salmonella enterica*, and *Serratia marcescens* (Sifri et al., 2003; Elkabti et al., 2018), as well as fungal pathogens such as *Cryptococcus neoformans* and *Candida albicans* (Madende et al., 2019), such that researchers have used them as models to study microbial pathogenesis and innate immunity. *C. elegans* can also be used in liquid assays and high-throughput screens, making them valuable models for use in the discovery of novel bioactive compounds (Anastassopoulou et al., 2011; Chen et al., 2021a). As such, we herein utilized a CRKP-infected *C. elegans* model system to evaluate the antimicrobial activity of antibacterial drugs commonly utilized in clinical contexts in an effort to identify drugs that may offer promise for the treatment of CRKP-infected patients.

## Materials and methods

### Carbapenem-resistant *Klebsiella pneumoniae*

Sequence type (ST) 11 KPC-2-producing CRKP (G type) is the most common CRKP isotype in our hospital (Liu et al., 2018), and was thus selected as the model strain for this study. This model strain was provided by the clinical laboratory department of our hospital. The KPC-2 gene was screened from these isolates and used to conduct a multilocus sequence typing (MLST) analysis. The genus and species of isolated microbes were identified using the VITEK 2 Compact system (bioMérieux, Marcy l’Étoile, France), with Microflex™ matrix-assisted laser desorption/ionization time-of-flight mass spectrometry (MALDI-TOF MS; Bruker Daltonics, Bremen, Germany) being used for further verification.

### Caenorhabditis elegans culture

Wild type *C. elegans* [N2, *Caenorhabditis* Genetics Center (CGC), University of Minnesota] were maintained in Nematode Growth Media supplemented with *Escherichia coli* OP50 (OP50, obtained from CGC) as a food source. Nematodes were raised at 20°C on NGM plates (1 mM CaCl_2_, 1 mM MgSO_4_, 5 g/ml cholesterol, 50 mM KH_2_PO_4_ pH 6.0, 25 mM NaCl, 1.7% agar, and 2.5 mg/ml peptone) with freshly added OP50. Synchronized nematodes in the L4 stage were used for all experiments.

### Bacterial culture

*E. coli* strain OP50 was grown in LB medium containing Streptomycin/ampicillin at 37°C with constant shaking overnight. ST11 CRKP strains were grown at 37°C without agitation in BBL Trypticase soy broth (TSB) for 16 h such that there were in the logarithmic phase of growth. Both bacteria were resuspended at 1.5 CFU/ml × 10^9^ CFU/ml, and aliquots of these bacteria were maintained at −80°C in beef bouillon with 10% glycerol (Statens Serum Institut).

### *Caenorhabditis elegans* synchronization

Nematode synchronization was conducted as in prior reports (Possik and Pause, 2015). Briefly, 20 gravid adult hermaphrodites were added to fresh NGM plates containing OP50. Nematodes were allowed to lay eggs for 6 h, after which a platinum wire nematode pick was utilized to remove the mothers. Eggs were allowed to hatch and grow at 20°C for 48°h, at which time the nematodes were in the L4 stage.

### Measurement of CRKP-infected nematode survival

OP50 and CRKP bacteria were grown as above, centrifuged, washed, and resuspended at target densities. The OP50 density was 1.5 CFU/ml × 10^9^ CFU/ml. Optimal CRKP infectious doses were identified by infecting *C. elegans* with 1.5 CFU/ml × 10^6^ CFU/ml, 1.5 CFU/ml × 10^7^ CFU/ml, and 1.5 CFU/ml × 10^8^ CFU/ml of CRKP microbes. Briefly, 600 synchronized L4 *C. elegans* were separated into four groups (*n* = 150/group) and added to 6-well NGM plates supplemented with 10 μl 5-Fluorouracil (20 μM) to inhibit nematode reproduction. Nematodes were then treated with 100 μl the three experimental CRKP concentrations (1.5 CFU/ml × 10^6^ CFU/ml, 1.5 CFU/ml × 10^7^ CFU/ml, and 1.5 CFU/ml × 10^8^ CFU/ml). Control nematodes were fed with 100 μl OP50. Plates were incubated at 20°C, and nematode death was monitored daily using a microscope. Experiments were repeated a minimum of three times.

### Drug preparation

Kirby-Bauer disk diffusion analyses revealed that CRKP isolates exhibited high resistance to almost all the drugs available in the laboratory, with particularly marked resistance to cephalosporins and carbapenems (Yao et al., 2021). In our study, the *in vivo* sensitivity of CRKP-infected *C. elegans* to 10 common antimicrobial agents including meropenem (MEM), imipenem (IPM), cefoperazone-sulbactam (SCF), amoxicillin-clavulanic acid (AMC), ceftazidime (CAZ), aztreonam (ATM), fosfomycin (FOT), amikacin (AK), tigecycline (TGC), and polymyxin B (PB) was investigated. All of these compounds were purchased from Sinopharm Chemical Reagent Co., Ltd. (Shanghai, China), and stock solutions for each were prepared using distilled water at concentrations of 10 mg/ml for MEM, 10 mg/ml for IPM, 120 mg/ml for SCF, 120 mg/ml for AMC, 40 mg/ml for CAZ, 40 mg/ml for ATM, 320 mg/ml for FOT, 100 mg/ml for AK, 20 mg/ml for TGC, and 10 mg/ml for PB.

### Drug sensitivity testing

The sensitivity of CRKP-infected nematodes to the 10 different target antibiotics was assessed in liquid media using a standard 96-well plate-based approach (Chen et al., 2021a). Briefly, synchronized L4 *C. elegans* were added into the individual wells of 96-well plates containing 100 μl of CRKP solution (1.5 CFU/ml × 10^8^ CFU/ml), after which appropriate doses of the selected antimicrobial agents were added based on the minimum inhibitory concentration (MIC) values calculated *in vitro* according to Clinical and Laboratory Standards Institute (CLSI) guidelines. Final concentrations of antimicrobial agents ranged from low to high (1X, 2X, 4X, 8X, and 16X MIC) as shown in Table 1. Nematodes were only fed 100 μl of OP50 culture as a control group, or were only given 100 μl of CRKP bacteria without any antimicrobial treatment as a negative treatment group. In total, 100 nematodes were added per well with five replicate samples per condition. Plates were incubated at 20°C for 12 h, and the number of surviving nematodes was counted after 2, 6, 8, and 12 h *via* microscopic examination. Experiments were repeated three times.

**TABLE 1:** Concentrations of 10 different antimicrobial drugs used for drug sensitivity testing.

Antibiotics	MIC (µg/ml)	2MIC (µg/ml)	4MIC (µg/ml)	8MIC (µg/ml)	16MIC (µg/ml)
MEM	2	4	8	16	32
IPM	2	4	8	16	32
SCF	24	48	96	192	384
AMC	24	48	96	192	384
CAZ	8	16	32	64	128
ATM	8	16	32	64	128
FOT	64	128	256	512	1024
AK	16	32	64	128	256
TGC	4	8	16	32	64
PB	2	4	8	16	32

MIC, minimum inhibitory concentration; MEM, meropenem; IPM, imipenem; SCF, cefoperazone-sulbactam; AMC, amoxicillin-clavulanic acid; CAZ, ceftazidime; ATM, aztreonam; FOT, fosfomycin; AK, amikacin; TGC, tigecycline; PB, polymyxin B.

## Determination of drug sensitivity of CRKP strain *in vitro* and *in vivo*

Ten CRKP strains samples were isolated from clinical specimens of hospitalized patients admitted to Xinhua Hospital in Shanghai, from May 2021 to December 2021. The sensitivity of CRKP strains to the ten different target agents were assessed *in vitro* and *in vivo*, respectively. In our study, *in vitro* antimicrobial susceptibility testing was performed using the K-B disk diffusion method by clinical laboratory department, and the results were expressed as susceptible (S), intermediate (I), or resistant (R) according to CLSI guidelines (Humphries et al., 2021). And *in vivo* antimicrobial susceptibility testing was performed using CRKP-infected nematode model according to the above steps, and the results were expressed as S, I or R based on the MIC of antibiotics and survival rate of nematodes protected by antibiotics (At 6 h, nematode survival rate ≥70% was considered effective). The thresholds of 50%–70% were commonly used in previous studies (Kong et al., 2014; Ganesh Kumar et al., 2019; Ahamefule et al., 2021), and we found that the drug sensitivity results obtained from the nematode model method using threshold of 70% were in good agreement with the results obtained from K-B method, according to the criteria in Table 2.

**TABLE 2:** The scope of drug sensitivity results of nematode model.

Antibiotics	MIC (µg/ml) Standard Explain		
	S	I	R
MEM	≤8	16	≥32
IPM	≤8	16	≥32
SCF	≤96	192	≥384
AMC	≤96	192	≥384
CAZ	≤32	64	≥128
ATM	≤32	64	≥128
FOT	≤64	128	≥256
AK	≤32	64	≥128
TGC	≤16	32	≥64
PB	≤8	16	≥32

MIC, minimum inhibitory concentration; MEM, meropenem; IPM, imipenem; SCF, cefoperazone-sulbactam; AMC, amoxicillin-clavulanic acid; CAZ, ceftazidime; ATM, aztreonam; FOT, fosfomycin; AK, amikacin; TGC, tigecycline; PB, polymyxin B.

### Statistical analysis

Data are means ± standard deviation (SD) from triplicate experiments. Kaplan-Meier survival curves were used to assess *C. elegans* survival and were analyzed using the Mantel-Cox log-rank test with Bonferroni’s correction for multiple testing using R version 4.1.0 (The R Foundation, Vienna, Austria). *p* < 0.05 was the threshold of statistical significance.

## Results

### CRKP-infected nematode model establishment

#### Analysis of *Caenorhabditis elegans* survival following infection with different CRKP doses

**FIGURE 1:**
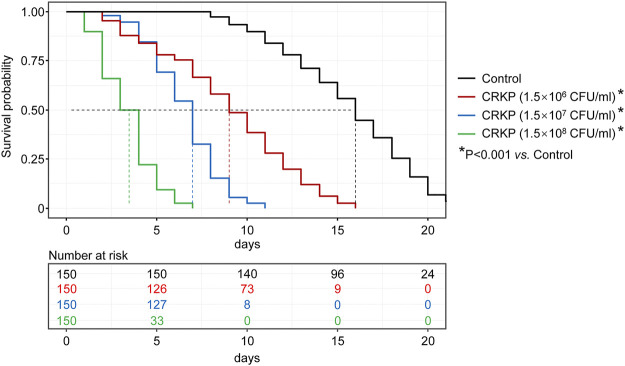
*C. elegans* survival curves following exposure to different CRKP concentrations. Nematodes in the control, 1.5 × 10^6^, 1.5 × 10^7^, and 1.5 × 10^8^ CFU/ml groups survived for 22, 15, 11, and 7 days, respectively, with median survival durations in these four respective groups of 16, 9, 7, and 3.5 days. Relative to control group, those in the three infection groups exhibited significantly impaired survival (*p* < 0.001).

To determine the optimal CRKP dose to use for our CRKP-infected *C. elegans* model system, the survival of these nematodes was monitored following infection with three tested CRKP doses (1.5 CFU/ml × 10^6^ CFU/ml, 1.5 CFU/ml × 10^7^ CFU/ml, and 1.5 CFU/ml × 10^8^ CFU/ml) was assessed (Figure 1). Nematodes in the control, 1.5 CFU/ml × 10^6^ CFU/ml, 1.5 CFU/ml × 10^7^ CFU/ml, and 1.5 CFU/ml × 10^8^ CFU/ml groups survived for 22, 15, 11, and 7 days, respectively, with median survival durations in these four respective groups of 16, 9, 7, and 3.5 days. Relative to control nematodes, those in the three infection groups exhibited significantly impaired survival (*p* < 0.001), with increasing CRKP doses resulting in a reduction in the time to 50% lethality (LT50).

To further highlight that CRKP infection with nematodes was dose-dependent, we additionally assessed the 7-days survival rates of nematodes in these infection groups, revealing survival rates of 66.7%, 15.3%, and 0% in the 1.5 CFU/ml × 10^6^ CFU/ml, 1.5 CFU/ml × 10^7^ CFU/ml, and 1.5 CFU/ml × 10^8^ CFU/ml groups, respectively. As such, these data indicate that CRKP bacteria can induce *C. elegans* death in a dose-dependent manner. In our study, in order to rapidly simulate a clinical infection model, and to realize the screening of sensitive antibiotics within the limited time of clinical treatment, we selected the highest tested CRKP dose (1.5 CFU/ml × 10^8^ CFU/ml) for use in subsequent studies.

### Drug sensitivity testing

#### Assessment of the antimicrobial activity of different drugs over time in CRKP-infected nematodes

**FIGURE 2:**
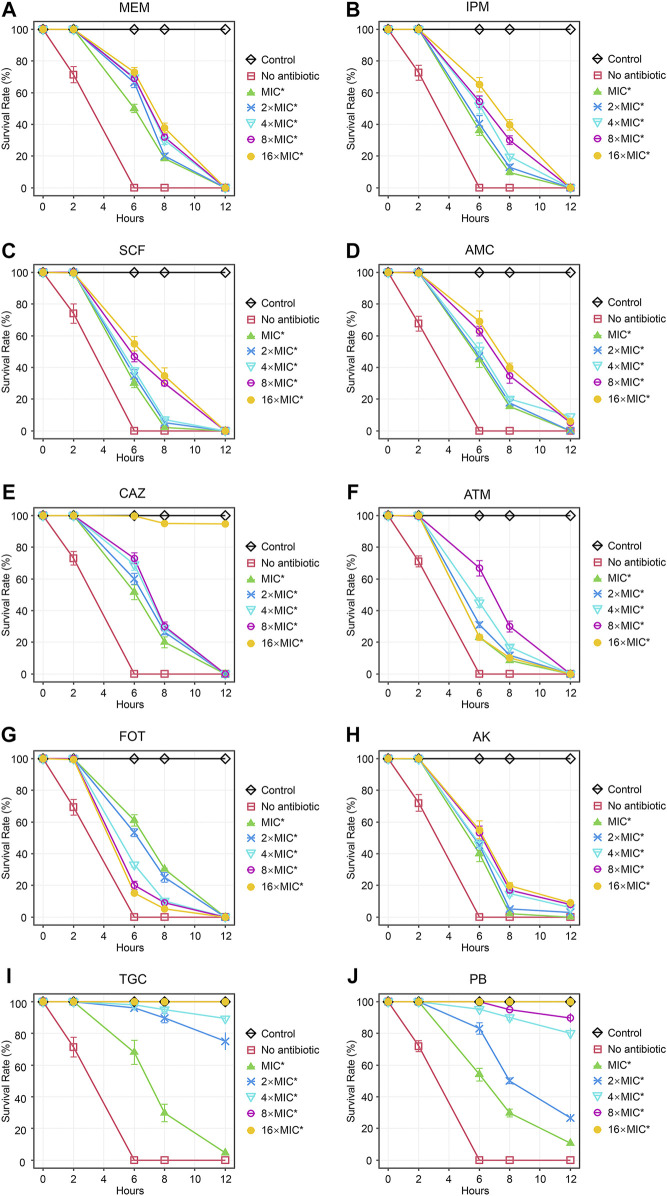
Time curve analyses of the protective efficacy of different concentrations of antibiotics in CRKP-infected *C. elegans.* Time curves were used to evaluate the time-dependent changes in the survival of CRKP-infected *C. elegans* treated with different concentrations of **(A)** MEM, **(B)** IPM, **(C)** SCF, **(D)** AMC, **(E)**, CAZ, **(F)** ATM, **(G)** FOT, **(H)** AK, **(I)** TGC, and **(J)** PB. Results are expressed as mean ± SD. Asterisks represent the significance of the difference in survival between the antibiotic-treated group and no antibiotic group (*p* < 0.001).

**FIGURE 3:**
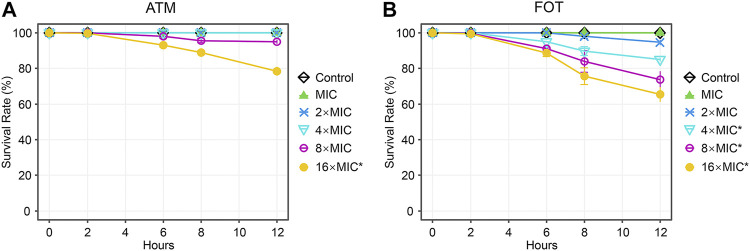
Time curve analyses of the toxicity of different concentrations of antibiotics in *C. elegans.* Time curves were used to evaluate the time-dependent changes in the survival in *C. elegans* treated with different concentrations of **(A)** ATM, **(B)** FOT. Results are expressed as mean ± SD. Asterisks represent the significance of the difference in survival between the antibiotic-treated group and control group (*p* < 0.001).

Next, we tested the antimicrobial efficacy of 10 common antibiotics including MEM (Figure 2A), IPM (Figure 2B), SCF (Figure 2C), AMC (Figure 2D), CAZ (Figure 2E), ATM (Figure 2F), FOT (Figure 2G), AK (Figure 2H), TGC (Figure 2I), and PB (Figure 2J) in our CRKP-infected *C. elegans* model system at the 0, 2, 6, 8, and 12 h post-treatment time points. Each drug was diluted from 1–16 × MIC (MIC, 2 × MIC, 4 × MIC, 8 × MIC, and 16 × MIC). At the 12 h time point, all animals in the control group were alive, whereas all animals in “no antibiotic” group died within 6 h. Survival rates of nematodes in the various antimicrobial treatment groups were higher with increasing concentrations. Most notably, PB and TGC exhibited robust antibacterial activity within 12 h, even at low tested concentrations (≥2 × MIC). At the 6 h time point, high concentrations of MEM (≥4 × MIC), CAZ (≥8 × MIC), TGC (≥2 × MIC), and PB (≥2 × MIC) all exhibited ≥70% antibacterial activity. The beneficial effects of FOT (≥4 × MIC) and ATM (≥16 × MIC) were compromised at high concentrations, with nematodes dying more rapidly than in the low concentration groups, suggesting that at higher doses the toxic effects of these compounds may outweigh the survival benefits attributable to their antimicrobial activity. Host toxic effect of the antibiotics on nematodes were evaluated, and results confirmed that FOT (≥4 × MIC) and ATM (≥16 × MIC) were toxic to nematodes at high concentrations (Figure 3). No other antibiotics exhibited any toxicity to the nematodes at the dose range we used.

As the duration of the study was extended, the apparent antimicrobial activity of these different antibiotics tended to decrease, with particularly notable declines for MEM, IPM, SCF, ATM, and FOT, all of which exhibited a loss of protective efficacy by the 12 h time point irrespective of concentration. However, high concentrations of TGC (≥8 × MIC) and PB (16 × MIC) retained 100% antibacterial activity at 12 h, and high doses of CAZ (≥16 × MIC), TGC (2 × MIC and 4 × MIC), and PB (4 × MIC and 8 × MIC) also exhibited substantial benefits to the survival of CRKP-infected nematodes at the 12 h time point.

### Drug sensitivity test results of CRKP strains *in vitro* and *in vivo*

Results of drug sensitivity of 10 samples of CRKP strains *in vitro* (K-B method) and *in vivo* (nematode model method) were shown in Table 3. K-B method suggested that CRKP was resistant to MEM and CAZ, while *C. elegans* model method suggested that some CRKP strains were intermediate to CAZ (4/10) and MEM (5/10). The sensitivity of CRKP to other drugs were consistent *in vivo* and *in vitro* excluding one that had an inconsistent response to FOT and another that had an inconsistent response to PB. Based on the above results, we preliminarily believe that nematode model can screen some antibacterial activities *in vivo* to a certain extent.

**TABLE 3:** Antimicrobial susceptibilities of 10 CRKP strains obtained from patients.

No.	Sample	Sex	Age (year)	Method	MEM	IPM	SCF	AMC	CAZ	ATM	FOT	AK	TGC	PB
1	Sputum	Male	77	K-B	R	R	R	R	R	R	S	S	S	S
				nematode	R	R	R	R	I	R	S	S	S	S
2	Sputum	Female	87	K-B	R	R	R	R	R	R	S	S	S	S
				nematode	I	R	R	R	R	R	S	S	S	S
3	Blood	Female	3	K-B	R	R	R	R	R	I	S	S	S	I
				nematode	I	R	R	R	I	I	S	S	S	S
4	Blood	Male	1	K-B	R	R	R	R	R	R	S	S	S	S
				nematode	I	R	R	R	R	R	S	S	S	S
5	Urine	Male	81	K-B	R	R	R	R	R	R	R	R	S	S
				nematode	I	R	R	R	I	R	I	R	S	S
6	Blood	Female	5	K-B	R	R	R	R	R	R	R	R	S	S
				nematode	R	R	R	R	R	R	R	R	S	S
7	Sputum	Male	53	K-B	R	R	R	R	R	R	S	R	S	S
				nematode	R	R	R	R	R	R	S	R	S	S
8	Blood	Male	76	K-B	R	R	R	R	R	R	S	R	S	S
				nematode	I	R	R	R	I	R	S	R	S	S
9	Sputum	Male	83	K-B	R	R	R	R	R	R	R	R	S	S
				nematode	R	R	R	R	R	R	R	R	S	S
10	Blood	Male	57	K-B	R	R	R	R	R	I	R	S	S	S
				nematode	R	R	R	R	R	I	R	S	S	S

The differences in the results obtained by the two methods are indicated by a gray background.

CRKP, carbapenem-resistant *Klebsiella pneumoniae*; K-B, Kirby-Bauer; MEM, meropenem; IPM, imipenem; SCF, cefoperazone-sulbactam; AMC, amoxicillin-clavulanic acid; CAZ, ceftazidime; ATM, aztreonam; FOT, fosfomycin; AK, amikacin; TGC, tigecycline; PB, polymyxin B.

### Typical clinical cases

To demonstrate the applicability of the nematode model method in clinical settings, two typical clinical cases are presented.

#### Case 1

A 77-year-old male with a history of type 2 diabetes mellitus, hypertension, and right upper lobectomy, presented in the respiratory department because of cough, expectoration, shortness of breath and fever for 15 days. He was febrile (39.0°C), with blood pressure of 125/80 mmHg, pulse rate of 80 bpm, and SpO_2_ of 95%. Laboratory tests were notable for a WBC of 14.27 × 10^9^/L with neutrophil ratio of 90.9%, CRP of 167 mg/L, and chest X-ray showed bilateral lung infiltrates. He was started on intravenous fluids, and dosed with broad-spectrum antibiotics, including MEM (1.0 g, Q8h, IVGTT) and AK (0.4 g, QD, IVGTT). After 5 days of treatment, fever was still present, and the clinical symptoms and signs were not significantly improved. Twice sputum cultures showed CRKP, which was resistant to MEM, and susceptible to AK, TGC, FOT and PB. However, *C. elegans* model method suggested that the CRKP strain was intermediate to CAZ. The antibiotic regimen was changed to CAZ (2 g, Q8h, IVGTT) combined with AK according to our test results. Three days later, temperature returned to normal, and infection symptoms improved significantly. He eventually recovered and was discharged after 19 days intensive treatment.

#### Case 2

A 3-year-old female child with a history of intrauterine distress and enterectomy. Her height and weight were 90 cm and 12.5 kg, respectively. She presented in pediatric gastroenterology department because of short bowel syndrome with severe malnutrition and enteritis. She was febrile (37.9°C), with blood pressure of 115/70 mmHg, pulse of 88 bpm, and SpO_2_ of 98%. Laboratory tests revealed a WBC of 12.22 × 10^9^/L with neutrophil ratio of 28.8%, and CRP of 8 mg/L. She was started on intravenous fluids and dosed with antibiotics cefuroxime (30 mg/kg Q8h IVGTT). Her condition deteriorated, and a CRKP strain was detected in the blood culture isolates on day 3 of hospitalization. *C. elegans* model method was performed and the results were available within 24 h, which suggested that strain was intermediate to MEM, CAZ, ATM, and susceptible to FOT, TGC, AK, and PB. Due to the urgency of the patient’s condition, antibiotic regimen was immediately changed to MEM (20 mg/kg, Q8h, IVGTT) combined with FOT (100 mg/kg, Q8h, IVGTT) according to the test results. Two days later, susceptibility tests performed with K-B method indicated that CRKP was resistant to MEM, intermediate to PB, but susceptible to FOT, TGC, and AK. The treatment regimen remains unchanged due to clinical improvement with decreased serum levels of acute-phase proteins such as CRP. Her conditions continued to improve over the following days, and CAZ (50 mg/kg, Q12h, IVGTT) was used as consolidation treatment. She was cured and discharged after 22 days of treatment.

## Discussion

*C. elegans* have long been used as a model system in a variety of research contexts owing to advantages including low costs and amenability to large-scale *in vivo* screening without the ethical concerns associated with the use of mammals in the context of drug testing (Ewbank and Zugasti, 2011). These nematodes have been proposed as an ideal model system for high-throughput screens of natural and synthetic drugs for activity against specific bacterial species (Sorathia and Rajadhyaksha, 2016), leading to their growing uptake in this research context. Kong et al., for example, established a *C. elegans*-*Staphylococcus aureus* liquid-based assay screening platform to identify antimicrobial agents with activity against *S. aureus* (Kong et al., 2014), while Joshi et al. similarly utilized these nematodes as a host for *Pseudomonas aeruginosa* to validate the *in vivo* antibacterial activity of their polyherbal “Panchvalkal” preparation (Joshi et al., 2019). Elkabti et al. further used a *C. elegans*-*Candida albicans* model system to screen for novel antifungal agents and to test both the efficacy and toxicity of these drugs (Elkabti et al., 2018). Ahamefule et al. used an Aspergillus fumigatus-infected *C. elegans* model for similar antifungal drug screening, efficacy analyses, and the assessment of host-pathogen interactions (Ahamefule et al., 2020). CRKP has emerged as a drug-resistant bacterial threat of growing concern owing to a dearth of effective treatments and rising CRKP infection incidence rates (Xu et al., 2017). *C. elegans* represent a particularly valuable model system for studies of human pathogens (Sorathia and Rajadhyaksha, 2016; Kim et al., 2017), and we thus established a *C. elegans* model method to facilitate the small-scale *in vivo* screening for antimicrobial agents with activity against CRKP.

For this study, 10 common clinical antimicrobial agents including MEM, IPM, SCF, AMC, CAZ, ATM, FOT, AK, TGC, and PB were selected for testing. In an *in vitro* Kirby-Bauer disk diffusion assay, all 10 antimicrobial agents exhibited therapeutic effects on sensitive KP, while most were resisted by CRKP, with the exception of PB, TGC, and FOT (van Duin et al., 2013; Zhang et al., 2019; Karakonstantis et al., 2020). These drugs are commonly used in combination with one another to treat CRKP in clinical settings, and we thus sought to better clarify the potential efficacy of these agents *in vivo* in our *C. elegans* model system in an effort to better identify drugs with promise for the treatment of CRKP-infected patients.

In our study, we found that higher CRKP concentrations were associated with reductions in LT50 values, median survival, and 7-days survival rates in infected nematodes. CRKP bacteria were thus able to promote *C. elegans* death in a dose- and time-dependent fashion. As such, to ensure the use of a rapid and stable model system, we used a high CRKP concentration (1.5 CFU/ml × 10^8^ CFU/ml) as a model for subsequent studies. After successful model establishment, we assessed the *in vivo* antimicrobial activity of different drugs over time in CRKP-infected nematodes. The results indicated that all tested antimicrobial agents had protective effects in CRKP-infected nematodes, revealing that higher survival rates were evident for nematodes treated with higher antimicrobial concentration doses. Notably, PB and TGC exhibited robust antimicrobial activity even after 12 h at low concentrations, and high doses of CAZ (≥16 × MIC) also exhibited potent efficacy as compared to other drugs. However, interestingly, at high doses, the beneficial effects of FOT (≥2 × MIC) and ATM (≥16 × MIC) treatment were no longer evident, with nematodes dying at a more rapid rate than following low-dose treatment, suggesting that the toxic side effects of these drugs may outweigh their antibacterial benefits at these doses (Breger et al., 2007).

In order to demonstrate the applicability of the nematode model method in clinical settings, ten CRKP strains samples were collected from clinical patients, and our *in vivo* sensitivity results were well consistent with that using the K-B method. Interestingly, a few different results were observed between the two methods. One of the reasons might be related to the complexity and diversity of the carbapenem resistance mechanisms of CRKP, including the production of carbapenemases, excessive expression of plasmid type AmpC enzyme and loss of fenestra proteins, quantity descent, loss or decreased affinity of carbapenem high affinity site PBP2, etc., (Di et al., 2018; Park et al., 2020; Lapp et al., 2021). Unfortunately, molecular analysis for the confirmation of genes encoding carbapenemase production was not performed due to technical limitations, which may in part explain the difference in the results. Another reason might be that *C. elegans* models, a whole-animal system where host-pathogen interactions are involved, can identify potential *in vivo* efficacy of antibiotics (Ewbank and Zugasti, 2011; Kim et al., 2017). This advantage of *C. elegans* model method was also illustrated in our cases. In case 1, CAZ showed moderate sensitivity to the CRKP strain in the nematode model, and patient obtained a significant improvement of the symptoms with the addition of CAZ, suggesting that CAZ possessed certain *in vivo* antibacterial activity against CRKP in case 1. In addition, the results using *C. elegans* model method could be obtained within 24 h, which was in marked contrast to the 48–72 h required by current methods. Previous studies have found that the time to appropriate antibiotic therapy is an independent predictor of 30-days mortality in patients with CRKP infection (Falcone et al., 2020; Chen et al., 2021a; Chen et al., 2021b), therefore clinicians should start appropriate antibiotic therapy as soon as possible. As shown in case 2, we obtained drug sensitivity results within 24 h using *C. elegans* model method, and selected the appropriate treatment regimen according to the results, with a successful outcome. Therefore, we believe that *C. elegans* model method has a good clinical application prospect and is worthy of further study.

## Conclusion

Overall, our study indicates that a *C. elegans* model system can be used to effectively screen compounds for their *in vivo* antimicrobial activity. As has been reported in the literatures (van Duin et al., 2013; Zhang et al., 2019; Yao et al., 2021), our data also suggested that PB and TGC represent promising antimicrobial agents for the treatment of CRKP, with high doses of CAZ (≥16 × MIC) serving as a possible alternative treatment. There are certain limitations to this approach, including the fact that we only screened a subset of clinically relevant antimicrobial agents, and we did not screen doses higher than 16 × MIC for those antibiotics that we did test. Meanwhile, large-scale samples are needed to determine the breakpoints of *C. elegans* model method. Future studies will be conducted to address these limitations. While nematodes are a promising model system for antimicrobial compound screening, they cannot fully recapitulate mammalian biology (Kong et al., 2016). For example, nematodes exhibit an efficient detoxification system that can constrain the potential identification of compounds that act by modifying host defense systems (Chen et al., 2021a). Even so, this *C. elegans* model holds great promise in the context of drug discovery and warrants further study.

## Data Availability

The original contributions presented in the study are included in the article/supplementary material, further inquiries can be directed to the corresponding authors.

## Abbreviations

AK, amikacin; AMC, amoxicillin-clavulanic acid; ATM, aztreonam; *C. elegans*, *Caenorhabditis elegans*; CAZ, ceftazidime; CRKP, carbapenem-resistant *Klebsiella pneumoniae*; FOT, fosfomycin; IPM, imipenem; K-B, Kirby-Bauer; MEM, meropenem; MIC, minimum inhibitory concentration; OP50, *Escherichia coli* OP50; PB, polymyxin B; SCF, cefoperazone-sulbactam; TGC, tigecycline.
